# Recent Advances in *bis*-Chalcone-Based Photoinitiators of Polymerization: From Mechanistic Investigations to Applications

**DOI:** 10.3390/molecules26113192

**Published:** 2021-05-26

**Authors:** Nicolas Giacoletto, Frédéric Dumur

**Affiliations:** Aix Marseille Univ, CNRS, ICR UMR 7273, F-13397 Marseille, France

**Keywords:** chalcone, ketone, photopolymerization, photosensitizers, Claisen-Schmidt condensation

## Abstract

Over the past several decades, photopolymerization has become an active research field, and the ongoing efforts to develop new photoinitiating systems are supported by the different applications in which this polymerization technique is involved—including dentistry, 3D and 4D printing, adhesives, and laser writing. In the search for new structures, *bis*-chalcones that combine two chalcones’ moieties within a unique structure were determined as being promising photosensitizers to initiate both the free-radical polymerization of acrylates and the cationic polymerization of epoxides. In this review, an overview of the different *bis*-chalcones reported to date is provided. Parallel to the mechanistic investigations aiming at elucidating the polymerization mechanisms, *bis*-chalcones-based photoinitiating systems were used for different applications, which are detailed in this review.

## 1. Introduction

**Figure 1:**
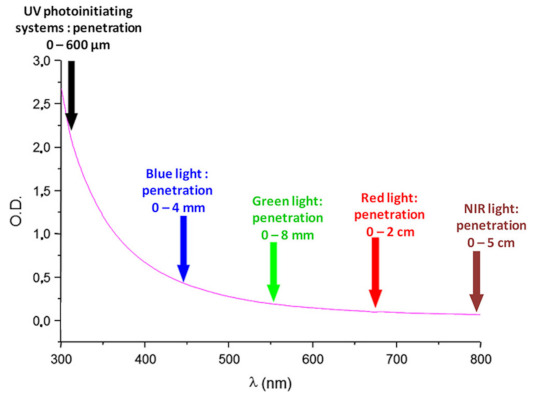
Light penetration in polystyrene latex with an average diameter of 112 nm. Reprinted with permission from Bonardi et al. [17]. Copyright 2018 American Chemical Society.

**Figure 2:**
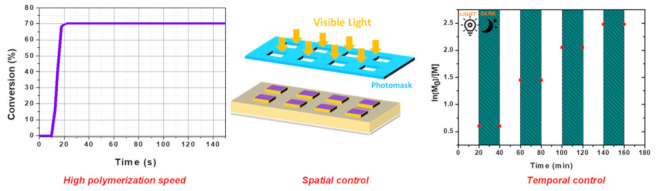
The different advantages of photopolymerization compared to traditional thermal polymerization.

Polymerization consists of converting a liquid resin into a solid, and different approaches can be used to obtain this result. As the most popular approach, the polymerization can be instigated by heat, and for this purpose, various thermal polymerization techniques have been developed over the years—such as ring-opening polymerization (ROP) [1,2], reversible addition–fragmentation chain-transfer (RAFT) polymerization [3], and nitroxide-mediated polymerization (NMP) [4,5,6]. Parallel to this, light can also be used to generate initiating species. While, historically, photopolymerization was mostly based on UV photoinitiating systems, UV light is now the focus of numerous safety concerns, such that a great deal of effort is now being devoted to developing visible light photoinitiating systems offering safer working conditions for the operator (no skin or eye damage) [7,8,9,10,11,12,13,14,15,16]. Furthermore, improved light penetration can be achieved in the visible range compared to that obtained with UV light, as shown in Figure 1. Indeed, if light penetration remains limited in the UV range (600 µm), a major improvement can be achieved with visible light, which can range between 4 mm and 5 cm, depending on the irradiation wavelength [17]. As a result of this, the scope of application of photopolymerization has been totally revolutionized, since the use of near-infrared light now allows for the polymerization of thick and filled samples, which is not achievable with UV photoinitiating systems [18,19,20]. The development of visible light photoinitiating systems is also supported by the easy access to cheap, compact, lightweight, and energy-saving irradiation sources such as light-emitting diodes (LEDs) [21,22]. Faced with this easy availability of LEDs with tunable irradiation wavelengths, the demand for photopolymerizable resins activable at these different wavelengths has similarly increased. In particular, numerous photoinitiating systems activable at 405 nm have been developed over the last several years—this wavelength being the wavelength currently in use for 3D printers [23,24,25,26,27,28]. Interest in photopolymerization is also supported by the different advantages this polymerization technique offers compared to traditional thermal polymerization, which can only be realized in solution. Thus, photopolymerization can be carried out in solvent-free conditions so that the release of volatile organic compounds (VOCs) can be advantageously avoided [29,30,31,32]. Natural compounds, photoinitiators, and monomers issued from renewable resources can also be used to elaborate photoinitiating systems and polymers, addressing the environmental impact and the toxicity issues raised by photopolymerization, and by polymerization more generally [33]. A spatial and a temporal control can also be obtained, meaning that the polymerization occurs only during the time the light is switched on, and only in the irradiated area (see Figure 2) [34,35]. The polymerization process can also be extremely fast, since it can be ended within a few seconds. This specificity is notably used advantageously with photopolymerizable glues and dental adhesives.

**Figure 3:**
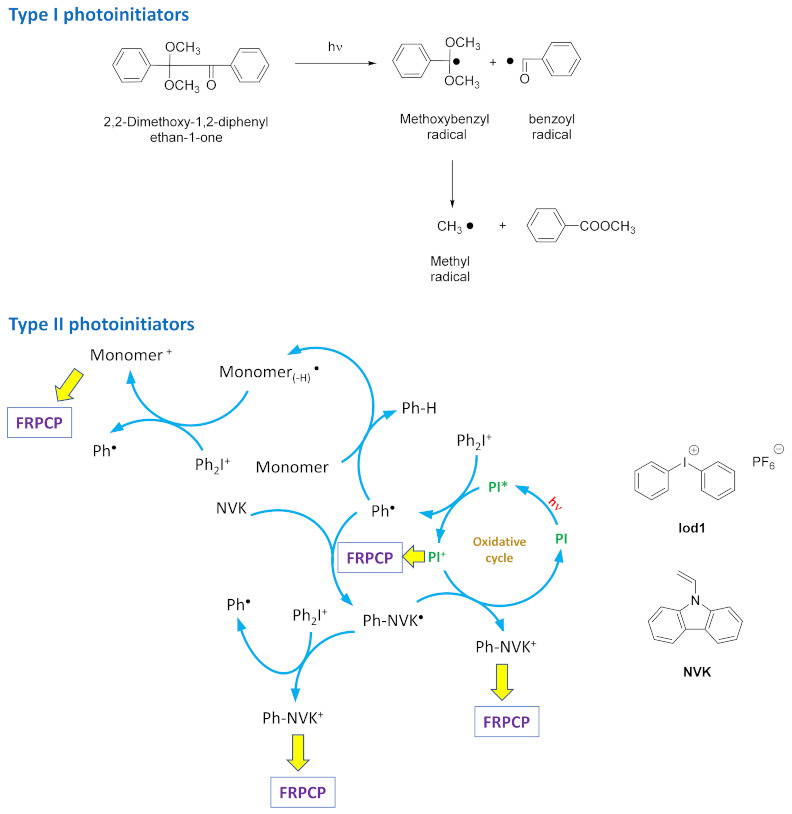
The two families of photoinitiators that have been developed in order to efficiently generate initiating radicals.

Considering that visible light photopolymerization can be activated between 400 and 800 nm, numerous dyes absorbing in the visible range have been proposed, as exemplified with acridine-1,8-diones [36,37,38], carbazoles [39,40,41,42,43,44], pyrenes [45,46,47,48,49,50], iridium complexes, [51,52,53,54,55,56,57,58,59], copper complexes [60,61,62,63,64,65,66,67,68,69,70], squaraines [71,72,73], camphorquinones [74,75], perylenes [76,77,78], iodonium salts [79,80,81], benzophenones [82,83,84,85,86,87], cyanines [88,89], diketopyrrolopyrroles [90,91,92], helicenes [93,94], naphthalimides [95,96,97,98,99,100,101,102,103,104,105,106,107], chalcones [108,109,110,111,112,113,114], iron complexes [115,116,117,118,119,120], chromones [121,122,123], thioxanthones [124,125,126,127], dihydroanthraquinones [128], porphyrins [129,130], zinc complexes [131], acridones [132,133], push–pull dyes [134,135,136,137,138,139,140,141,142,143,144,145], phenothiazines [146], coumarins [147,148,149,150,151,152,153], flavones [154], 2,3-diphenylquinoxaline derivatives [155], and cyclohexanones [156,157,158,159]. With the aim of generating initiating species, two distinct families of photoinitiators can be distinguished: The first family, type I photoinitiators, consists of molecules that can be photochemically cleaved upon excitation. The advantage of this strategy is that only a single component is necessary to generate the initiating radicals, so the migratability of potential side products within the polymer is considerably reduced. As shown in Figure 3 with 2,2-dimethoxy-1,2-diphenylethan-1-one, upon photoexcitation, a methoxybenzyl and a benzoyl radical are simultaneously formed, improving the efficiency of the initiating step. Additionally, the two radicals can be connected to the polymer chain under growth so that no migratable residue remains within the polymer network, addressing the potential toxicity issue of the photoinitiating systems. However, while this approach is appealing, the availability of visible Type I photoinitiators remains limited, and most of the benchmark Type I photoinitiators are UV photoinitiators [160,161,162]. As a drawback, Type I photoinitiators are irreversibly consumed during the polymerization process, and so the concentration of radicals irreversibly decreases over time. Conversely, Type II photoinitiators are typically dyes absorbing in the visible range, which act as photosensitizers for UV photoinitiators. Upon photoinduced electron transfer from the excited photosensitizer towards the UV photoinitiator, initiating radicals can be generated [163]. As the most widely used UV photoinitiators, onium salts, and notably iodonium salts, which are easily accessible from various commercial sources can be cited as relevant examples [164,165,166,167]. Considering that dyes act as photosensitizers for UV photoinitiators, two-component or three-component photoinitiating systems are typically developed with Type II photoinitiators.

As shown in Figure 3, upon excitation of the photoinitiator with a light of an appropriate wavelength, a photoinduced electron transfer in the excited state can occur with the iodonium salt, generating phenyl radicals Ph^●^. These radicals can typically initiate the free-radical polymerization of acrylates. However, in these conditions, the consumption of the photosensitizer is irreversible, affecting the efficiency of the system. This drawback can be addressed by the addition of a third component—generally, a sacrificial amine that will be in charge of reducing the oxidized photosensitizer, and which can be introduced to the photocurable resin. If *N*-vinylcarbazole (NVK) is used, this carbazole can react with the phenyl radical Ph^●^, generating Ph–NVK^●^, which is a radical more reactive than the initial Ph^●^ [168]. By reacting with the oxidized photosensitizer and regenerating the photosensitizer at its initial redox state, Ph–NVK^●^ can be converted into a Ph–NVK^+^ cation, capable of initiating the cationic polymerization of epoxides by means of free-radical-promoted cationic polymerization (FRPCP). Therefore, using these three-component systems, the concomitant polymerization of acrylates and epoxides can be simultaneously obtained, enabling access to interpenetrated polymer networks (IPN) [169,170,171,172]. The photoinitiating systems are also catalytic if three-component systems are used, the regeneration of photoinitiators enabling the system to drastically reduce its content [173,174,175]. Considering that the photosensitizer is the key element of these two- and three-component photoinitiating systems, numerous structures have been examined. In this field, chalcones are dyes that can be naturally found in numerous vegetables and flowers [176,177,178]. Chalcones can also be easily obtained via a Claisen-Schmidt condensation. Considering their ease of synthesis, their strong absorption in the visible range, and their well-established biological activities, chalcones were investigated for applications ranging from medicine [179] to solar cell applications [180,181], organic light-emitting diodes [182], and organogels [183]. Among chalcones, *bis*-chalcones—which can be obtained via a Claisen-Schmidt condensation of aldehydes with cyclic aliphatic ketones in basic conditions—have been less studied in the literature than *mono*-chalcones [109,184]. Moreover, these structures remain of interest, especially for photopolymerization. Indeed, by increasing the molecular weight of photoinitiators, their migratability within the polymer network can be drastically reduced. These dyes also possess an extended conjugation compared to *mono*-chalcones; thus, these photosensitizers can transfer an electron towards an electron acceptor more easily in the excited state.

It should be noted that, in the past, chalcones have been investigated in photopolymerization, but as molecules capable of initiating [2 + 2] cycloaddition reactions [185]. However, in this case, chalcones were acting in the dual role of monomers and sensitizers, and not as sensitizers capable of initiating the polymerization of acrylates or epoxides. In this review, an overview of the different *bis*-chalcones reported to date as photoinitiators of polymerization is provided. Although the initial reports were devoted to investigating the photochemical mechanism of initiation rapidly, potential applications of these *bis*-chalcone-based photoinitiating systems have been examined. Comparisons with reference compounds will also be established in order to demonstrate the potential of these new structures for photoinitiation.

## 2. The Different Synthetic Routes to *bis*-Chalcones

**Scheme 1:**
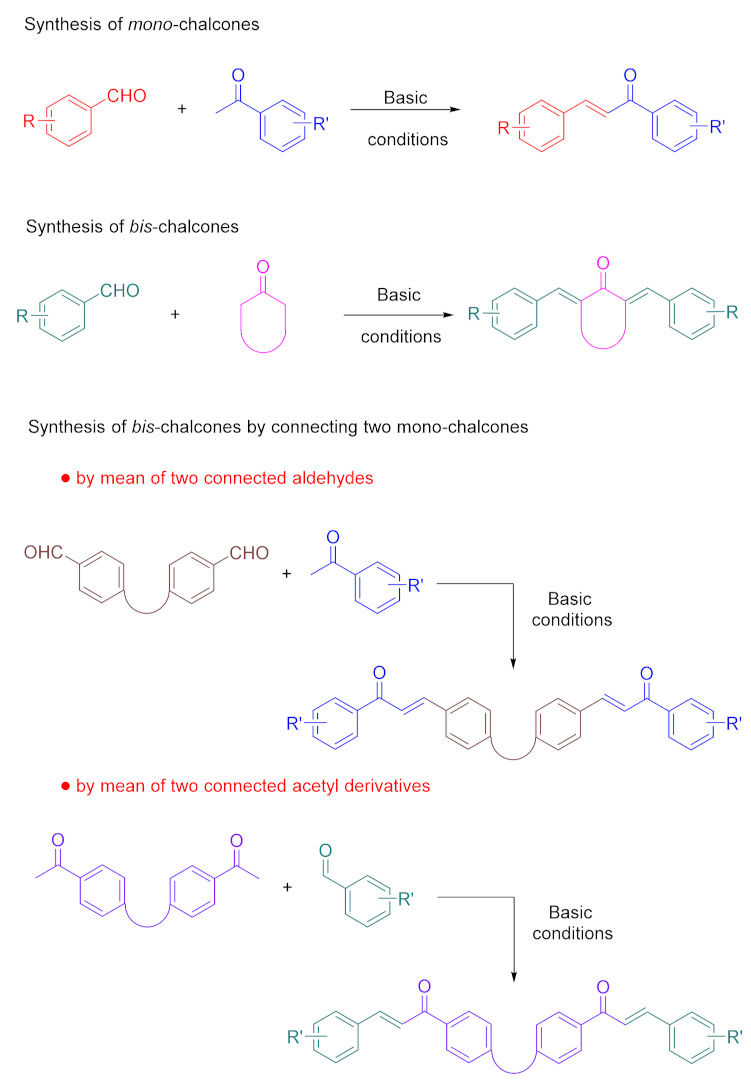
Synthetic routes to *mono-* and *bis*-chalcones; and *bis*-chalcones obtained by connecting two *mono*-chalcones.

*Bis*-chalcones have recently been studied as photoinitiators of polymerization, and three different strategies have been developed to provide access to these structures. Notably, chalcones can be easily obtained by means of a Claisen-Schmidt condensation between an aldehyde and an acetophenone, in accordance with the reaction depicted in Scheme 1 [186,187,188,189,190,191,192,193,194,195]. No major differences can be found from the synthetic viewpoint between *mono-* and *bis*-chalcones, except that two equivalents of aldehydes have to be used in the case of *bis*-chalcones, comprising a central cyclic ketone. In addition to this first strategy based on cyclic ketones, a second approach can consist of connecting two *mono*-chalcones together. In this aim, two connected aldehydes or two connected acetophenones can be used to form *bis*-chalcones.

**Figure 4:**
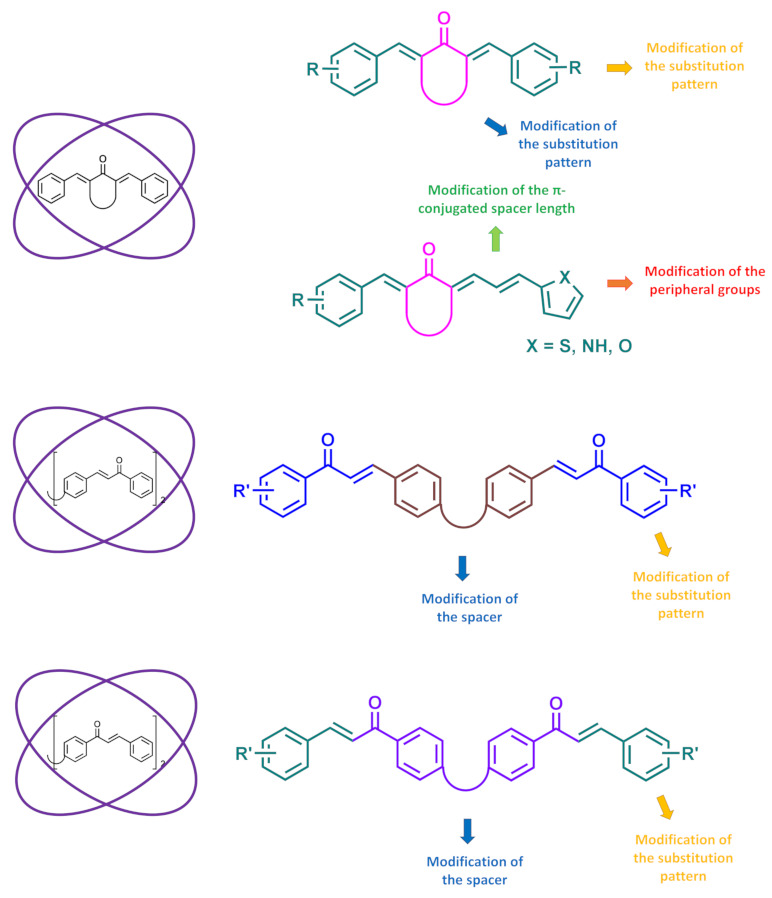
The different chemical modifications enabling the efficient tuning of the absorption spectra of *bis*-chalcones.

Based on the synthetic approach used to provide access to these structures, numerous modifications of the chalcone scaffold can be envisioned, such as a modification of the peripheral groups, the substitution pattern of the central cyclic aliphatic ketone, or the spacer introduced between the central core and the peripheral groups (see Figure 4). In the same spirit, *bis*-chalcones can be obtained via the condensation of acetophenones on *bis*-aldehydes. Aldehydes can also be condensed onto *bis*-acetophenones. Here, again, this strategy is highly versatile; since the spacer is used to connect the two chalcones, the substitution pattern of the chalcones can be easily tuned. Overall, the connection of two chalcones together enables a similar effect on the migratability of these macrophotoinitiators.

## 3. *Bis*-Chalcones as Photoinitiators

### 3.1. Bis-Chalcones Based on Cyclic Aliphatic Ketones

**Figure 5:**
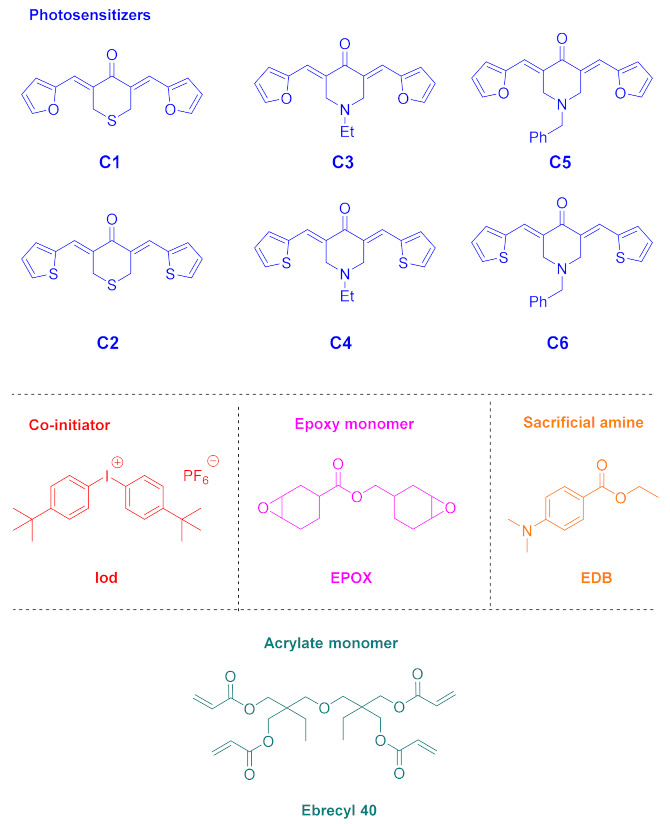
Chemical structures of C1–C6, the monomers, and the different additives.

**Figure 6:**
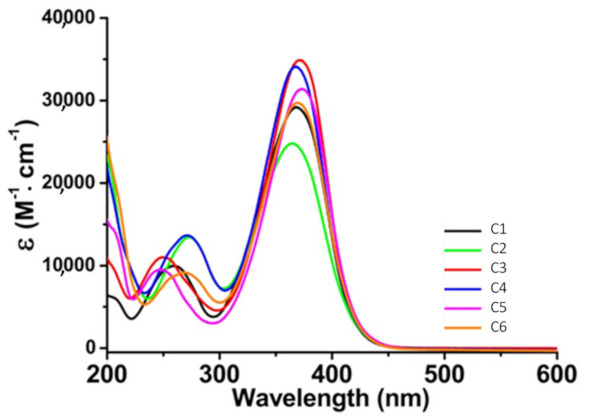
UV-visible absorption spectra of C1–C6 in acetonitrile. Reproduced from [197] with permission from The Royal Society of Chemistry.

While *mono*-chalcones had been tested as early as 2014 as versatile photoinitiators for the free radical, cationic, and thiol-ene polymerizations of various monomers [196], the first report mentioning the use of *bis*-chalcones as photoinitiators of polymerization was by Lalevée et al. in 2020, wherein a series of six chalcones (C1–C6) was examined (see Figure 5) [197]. Interestingly, for this series of six *bis*-chalcones, two peripheral groups were selected—namely, pyrroles and thiophenes. Indeed, these two groups are well known to exhibit low oxidation potential [198,199,200]. Three different cyclic ketones were also used as the central cores—namely, *N*-ethylpiperidinone, *N*-benzylpiperidinone, and thiopyranone. From the absorption viewpoint, almost no difference was found between the absorption spectra of C1–C6 (see Figure 6 and Table 1). Indeed, absorption maxima ranging between 365 nm for C2 and 372 nm for C5 were determined using UV-visible absorption spectroscopy. However, C3 and C4 bearing *N*-ethylpiperidinone as the central core showed the highest molar extinction coefficients. At 405 nm, major differences could be found between the different dyes. Thus, if molar extinction coefficients lower than 100,000 M^−1^·cm^−1^ were determined for C1 and C2 (9740 and 7980 M^−1^·cm^−1^) at 405 nm, molar extinction coefficients close to 120,000 M^−1^·cm^−1^ could be determined for C3 and C5 at 405 nm. It should be noted that for all of these dyes, the molar extinction coefficients at 405 nm were greatly lowered compared to their absorption maxima located around 370 nm. Even if the absorption obtained at 405 nm only constitutes the edges of the absorption bands, this absorption and the molar extinction coefficients at these positions remain sufficient to initiate a polymerization process.

Considering their absorptions at 405 nm, all of the dyes could be tested as photoinitiators at this wavelength. In order to get high monomer conversions, the different dyes were used in three-component systems, enabling *bis*-chalcones to be regenerated, and thus, to be introduced in catalytic amounts to the photoinitiating system. Using this strategy, an amount as low as 0.1 wt% could be used without adversely affecting the monomer conversion. While using the three-component chalcone/amine (EDB)/Iod (0.1%/2%/2%, *w*/*w*/*w*) photoinitiating systems (where EDB and Iod stand for ethyl dimethylaminobenzoate and 4,4′′-di-*tert*-butyldiphenyliodonium hexafluorophosphate, respectively), upon irradiation at 405 nm with an LED for 400 s, the best monomer conversions for thick films were obtained for C3 (94%) and C5 (90%), varying by the substitution pattern of the central piperidinone core (ethyl or benzyl groups) (see Table 2). The decrease in monomer conversion determined for the C5-based photoinitiating system was attributed to the bulkiness of the benzyl group, providing a less densified polymer network. Conversely, all of the chalcones containing a sulfur atom in their structures furnished only low monomer conversions—lower than 30% after 400 s of irradiation. The lowest monomer conversions were obtained for C2 and C4 (24% conversion after 400 s of irradiation for both of the two dyes). Comparisons between the monomer conversions obtained with C3 (94%) and C4 (24%) revealed the detrimental effect of thiophene moiety on the photoinitiating ability of *bis*-chalcones, with the two dyes only differing by their peripheral groups.

An opposite trend was found for the polymerization of thin films. Indeed, in this last case, the C4-based photoinitiating system proved to be the most efficient one, enabling a monomer conversion rate of 81%. Although the C3-based three-component system could still initiate a polymerization process, the lowest monomer conversion rate in thin films was obtained with this system, peaking at 55%. This therefore clearly demonstrates the dramatic influence of the substitution pattern, as well as the choice of the central ketones, on the photoinitiating ability.

**Figure 7:**
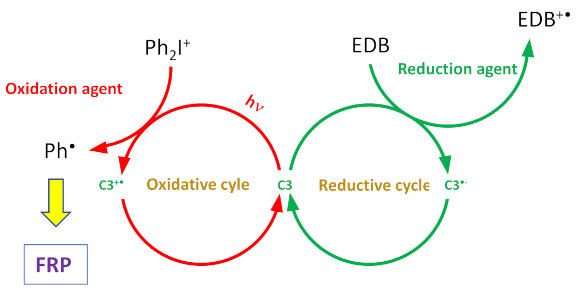
The dual role of C3 in three-component photoinitiating systems.

Considering that C3 could initiate polymerization in thick and in thin films, the polymerization mechanism was investigated using this dye. Photolysis experiments conducted in solution revealed C3 to efficiently interact with both the amine and the iodonium salt. Notably, a fast decrease in optical density was found upon irradiation at 405 nm for the two-component C3/amine and C3/Iod systems in acetonitrile. The ability of C3 to interact with reductive and oxidative processes was thus demonstrated (see Figure 7).

**Figure 8:**
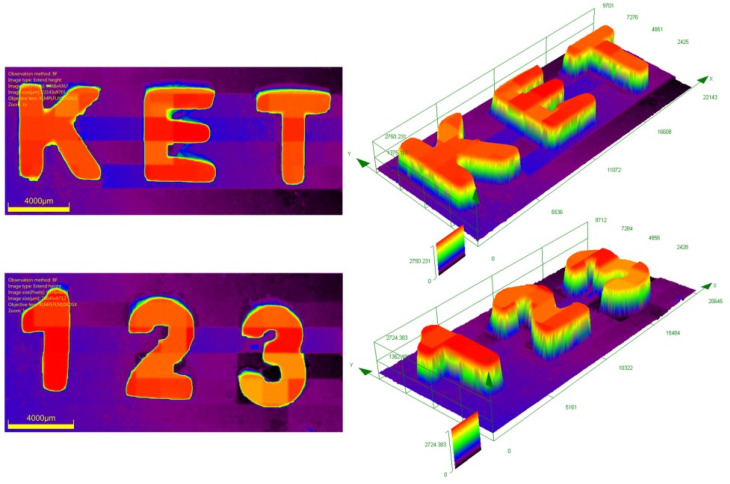
3D patterns obtained during laser writing experiments using the C3-based three-component system. Reprinted from [197] Copyright (2020), with permission from Elsevier.

Interestingly, C3 could also initiate the cationic polymerization of (3,4-epoxycyclohexane)methyl 3,4-epoxycyclo-hexylcarboxylate (EPOX) using the two-component C3/Iod (0.1%/2%, *w*/*w*) system. After 400 s of irradiation at 405 nm, a monomer conversion of 50% could be obtained with an LED (I = 110 mW/cm^2^). Finally, in light of the remarkable polymerization profiles obtained with C3, laser writing experiments were carried out with a laser (I = 100 mW/cm^2^) emitting at 405 nm. As anticipated, 3D patterns with a high spatial resolution (100 µm) were obtained, and a 2-cm-length pattern could be polymerized within two minutes (see Figure 8). Conversely, in the same conditions, no 3D patterns could be obtained using the C4-based three-component photoinitiating system, consistent with the results obtained during the mechanistic investigations. In this last case, only a polymerization at the surface of the 3D patterns could be obtained.

**Figure 9:**
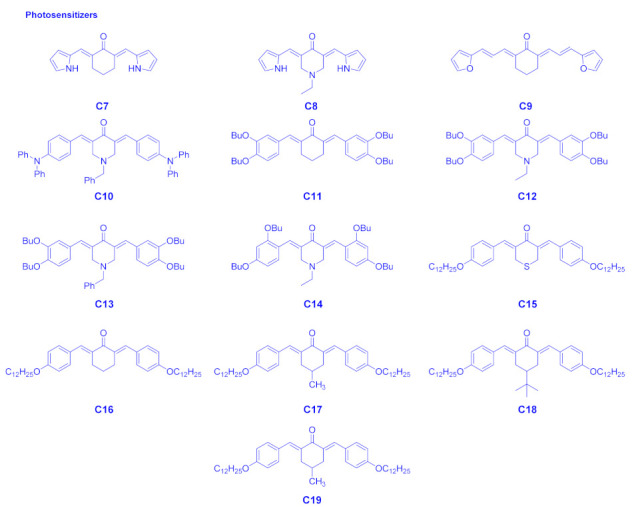
Chemical structures of C7–C19.

Following this initial work, another series of 13 *bis*-chalcones based on 6 different central cyclohexanones was examined in similar conditions to those used for the previous series (see Figure 9) [113]. From a synthetic viewpoint, all of these chalcones were obtained via a Claisen-Schmidt condensation, in a one-step reaction using aq. 40% KOH as the base, and the different dyes could be obtained with reaction yields ranging from 78% for C14 to 95% for C8. Ease of synthesis of such photoinitiators is of crucial interest from an industrial viewpoint, considering that most of the reagents (cyclohexanone derivatives, benzaldehyde derivatives, etc.) are cheap and commercially available. In this series, two groups of dyes could be identified.

Thus, while C7–C10 absorbed in the visible range (405 nm for C8 and C9, 416 nm for C7, and 434 nm for C10), conversely, absorptions of C11–C19 remained centered in the UV range, with absorption maxima ranging from 350 nm for C15 and C16 to 375 nm for C12–C14 (see Table 3). However, a sufficient absorption at 405 nm could be determined for all dyes so that the polymerization experiments could be carried out.

**Figure 10:**
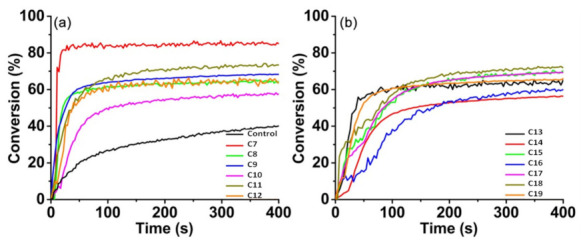
Polymerization profiles obtained using the three-component chalcone/amine/Iod (0.1%/2%/2%, *w*/*w*/*w*) system for C7–C12 (**a**) and C13–C19 (**b**); LED at 405 nm (110 mW/cm^2^), in laminate. Control experiment: amine/Iod (2%/2%, *w*/*w*). Reprinted with permission from John Wiley & Sons, Inc. Copyright © [113].

Photopolymerization experiments conducted with the two-component chalcone/Iod (0.1%/2%, *w*/*w*) and chalcone/amine (0.1%/2%, *w*/*w*) revealed that the conversions of Ebecryl 40 with the different photoinitiating systems remained limited—lower than 55% and 40%, respectively. Similarly, control experiments performed with the different chalcones alone (0.1% *w*) revealed the monomer conversion to be low, peaking at 20%. Conversely, no polymerization could be initiated with EDB or Iod alone. While using the three-component chalcone/amine/Iod (0.1%/2%/2%, *w*/*w*/*w*) system, a significant enhancement of the monomer conversion could be evidenced, demonstrating the necessity of mixing the three components together in order to form an efficient photoinitiating system (see Figure 10).

Thus, monomer conversions ranging from 56% for C14 to 85% for C7 were determined after irradiation with an LED at 405 nm for 400 s in laminate (see Table 4). Among the different photoinitiating systems investigated, the three-component system based on C7 clearly outperformed the others, since an improvement of the monomer conversion of at least 10% could be obtained with this *bis*-chalcone. As shown in Figure 10, the different systems were nevertheless reactive, since the oxygen inhibition could be efficiently overcome. All of the polymerizations started immediately after the light was switched on, and this feature is characteristic of highly reactive photoinitiating systems [201,202,203,204]. While examining the influence of the peripheral groups, high monomer conversions could be obtained while using pyrrole as the peripheral group. Conversely, the lowest monomer conversions were obtained using the triphenylamine or 2,4-dibutoxyphenyl groups. In these two cases, monomer conversions peaking at 56% could be obtained using each of the two three-component systems. Interestingly, comparisons of the monomer conversions obtained between 2,4-dibutoxyphenyl- and 3,4-dibutoxyphenyl-substituted chalcones (C12 and C14) revealed an improvement of the conversion of nearly 10% with the 3,4-dibutoxyphenyl-substituted chalcone compared to the 2,4-dibutoxyphenyl-substituted chalcone, whereas the two chalcones exhibited similar absorption characteristics (absorption maxima, molar extinction coefficients). Therefore, the influence of the substitution pattern was clearly demonstrated. While examining the respective absorptions of the different chalcones at 405 nm, no direct correlations between absorption and monomer conversion could be established. Other parameters, such as the rate constants of interaction of the chalcones with the different additives, and the excited state lifetime, also have to be taken into account. Notably, steady-state photolysis experiments revealed a consumption of 78% for C7 after 10 min of irradiation, while irradiating an acetonitrile solution containing C7 in combination with the iodonium salt. Conversely, under the same conditions, a consumption of only 32% was observed for C8, demonstrating that the photoinitiating ability of C7 was directly related to its higher photochemical reactivity with the iodonium salt, compared to C8–C19.

**Figure 11:**
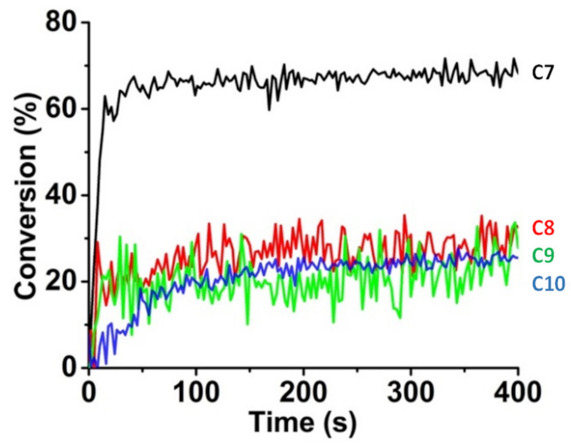
Polymerization profiles of EPOX obtained with two-component chalcone/Iod (0.1%/2%, *w*/*w*) systems upon irradiation at 405 nm with an LED. Reprinted with permission from John Wiley & Sons, Inc. Copyright © [113].

The two-component C7/Iod (0.1%/2%, *w*/*w*) system also proved to be promising for initiating the cationic polymerization of epoxides. Thus, an EPOX conversion of 70% could be obtained with the C7/Iod (0.1%/2%, *w*/*w*) system upon irradiation at 405 nm with an LED, whereas the EPOX conversions were lower than 30% for the C8–C10-based photoinitiating systems (see Figure 11). The reactivity of the photoinitiating systems was highly dependent on the rate constants of interaction with the additives. Thus, while the photolysis experiments with the two-component C7/amine and C8/amine systems in acetonitrile revealed a consumption of the ketones of 11% and 12%, respectively, after 10 min of irradiation at 405 nm, higher declines were obtained with the C7/Iod and the C8/Iod systems, reaching 78% and 32%, respectively. Therefore, the higher photoreactivity of C7 compared to that of C8 is directly linked to the rate constant of interaction with the additives.

Similarly to that suggested for the first series of *bis*-chalcones, and on the basis of the photolysis experiments, an oxidation–reduction photochemical mechanism could be proposed to support the monomer conversions. These conclusions were confirmed by the fluorescence quenching experiments performed in acetonitrile. Determination of the electron transfer quantum yields revealed the C7/Iod system to exhibit a higher value (0.923) than that determined for the C8/Iod system (0.899).

**Figure 12:**
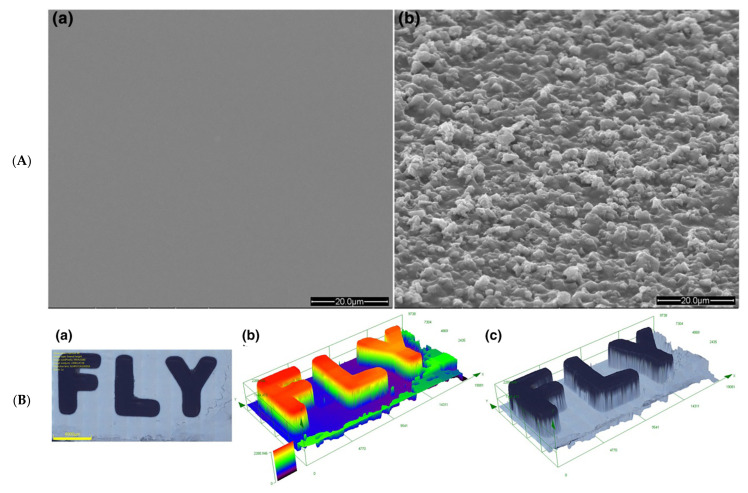
(**A**): SEM images of Ebecryl 40-based polymer, with (**a**) and without (**b**) silica fillers. (**B**): Images (**a**) (top view) and (**b**,**c**) (lateral view) obtained by numerical optical microscopy of 3D patterns obtained from laser writing experiments for resins containing 20% fillers, C7/amine/Iod (0.1%/2%/2%, *w*/*w*/*w*), 405 nm, 110 mW/cm^2^. Reprinted with permission from John Wiley & Sons, Inc. Copyright © [113].

Due to the high reactivity of the chalcone C7-based three-component system, the access to composites was thus examined. Indeed, the elaboration of composites remains a challenge with visible light photoinitiating systems, due to a limited light penetration and internal filter effects [205,206]. While introducing 20% weight of silica filler to Ebecryl 40, a severe limitation of the light penetration was demonstrated, as a light penetration of only 1.2 mm within the resin could be determined at 405 nm. By laser writing, 3D patterns exhibiting a remarkable spatial resolution could be obtained. An excellent dispersion of the fillers within the polymers could also be demonstrated by optical microscopy (see Figure 12).

**Figure 13:**
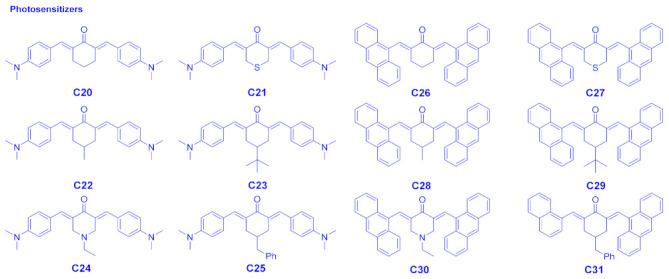
Chemical structures of C20–C31.

**Figure 14:**
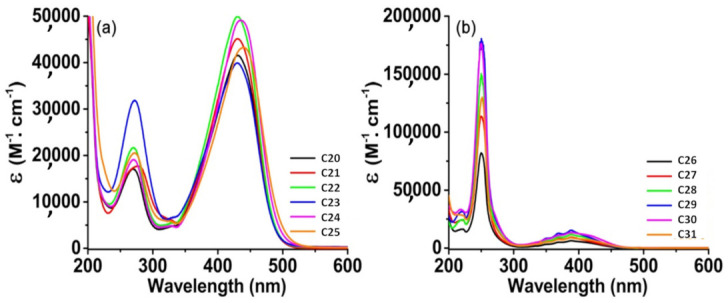
UV-visible absorption spectra of C20–C25 (**a**) and C26–C31 (**b**) in acetonitrile. Reproduced from [159] with permission from The Royal Society of Chemistry.

**Figure 15:**
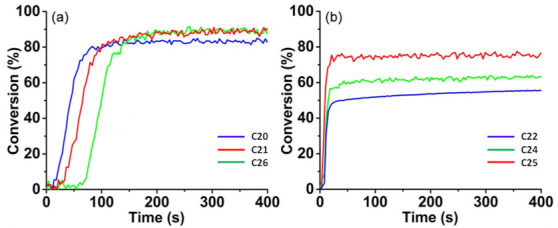
Photopolymerization profiles of TA, LED at 405 nm, using the three-component chalcone/amine/Iod (0.1%/2%/2%, *w*/*w*/*w*) photoinitiating systems: in thick films (1.4 mm) (**a**), and in thin films (25 μm) (**b**). Reproduced from [159] with permission from The Royal Society of Chemistry.

Peripheral groups of *bis*-chalcones can greatly influence the absorption spectra of dyes, and as such, a wide range of substituents has been examined over the years. Within a year, no less than 30 dyes differing by the peripheral groups have been proposed as photosensitizers, demonstrating the huge interest of photopolymerists in this family of compounds. Notably, the introduction of *para*-dimethylamino groups in C20–C25 enabled the development of dyes with absorption maxima ranging from 430 nm for C20, C21, and C23 to 436 nm for C24 and 440 nm for C25 (see Figure 13 and Figure 14 and Table 5) [159]. Concerning the molar extinction coefficients, the substitution pattern of the central cyclohexanone did not greatly influence the absorption properties, since molar extinction coefficients ranging between 40,000 M^−1^·cm^−1^ for C23 and 49,900 M^−1^·cm^−1^ for C22 could be determined in acetonitrile. A less intense absorption band was also detected in the UV range. A totally different situation was found for C26–C31. As shown in Figure 13 and Figure 14, an intense absorption band was detected at 250 nm for all anthracene-based chalcones, whereas an intramolecular charge-transfer band could be detected for all dyes, with absorption maxima located in the UV range. Moreover, the ICT bands were broad, extending from 300 to 450 nm, such that a sufficient absorption could be found at 405 nm for performing photopolymerization experiments. However, a threefold reduction of the molar extinction coefficients could be determined at 405 nm for anthracene-based chalcones compared to dimethylaminophenyl-based chalcones. When tested as photosensitizers for three-component chalcone/amine/Iod (0.1%/2%/2%, *w*/*w*/*w*) systems, no direct correlation between the molar extinction coefficient at 405 nm and final monomer conversions could be established. Indeed, if the dimethylaminophenyl-based chalcones produced higher monomer conversions in thick films, the opposite situation was found in thin films, with the anthracene-based chalcones outperforming the dimethylaminophenyl-based chalcones (see Table 6 and Figure 15). However, shorter inhibition times and steeper slopes could be observed for the polymerization curves obtained with the dimethylaminophenyl-based chalcones (see Figure 15).

**Figure 16:**
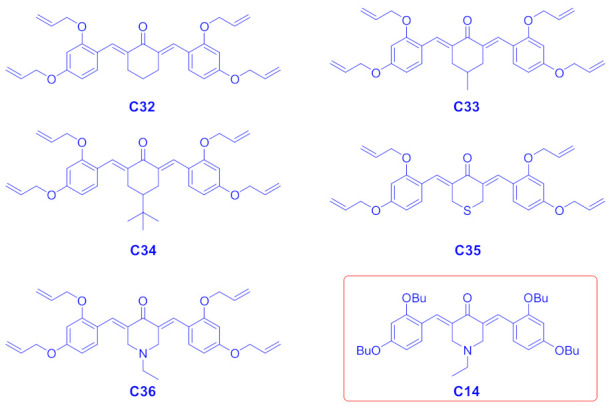
Chemical structures of crosslinkable chalcones C32–C36.

The extractability and migratability of photoinitiators is a major issue that can drastically affect future uses of polymers. In this context, in order to reduce the extractability of dyes, polymerizable groups can be introduced, as exemplified with C32–C36; for comparison, C14 [113], previously studied, was used as a non-crosslinkable chalcone (see Figure 16) [110].

The comparison between the crosslinkable chalcone C36 and its non-crosslinkable version C14 revealed the remarkable migration stability of C36 compared to C14. Notably, for the polymers obtained with the three-component chalcone/amine/Iod (0.1%/2%/2%, *w*/*w*/*w*) photoinitiating systems, examination of the migratability of dyes in acetonitrile revealed that only 0.9% of the initial chalcone C36 could be extracted, as opposed to 8.3% for chalcone C14. Therefore, by introducing a crosslinkable group, a ninefold reduction of the migratability could be obtained.

**Figure 17:**
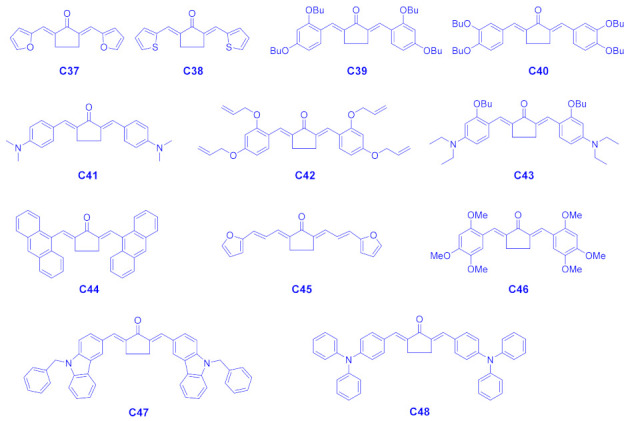
Chemical structures of C37–C48.

Recently, a series of 12 *bis*-chalcones based on cyclopentanone was proposed, with similar peripheral groups to those used for the design of cyclohexanone-based chalcones (see Figure 17) [207].

**Figure 18:**
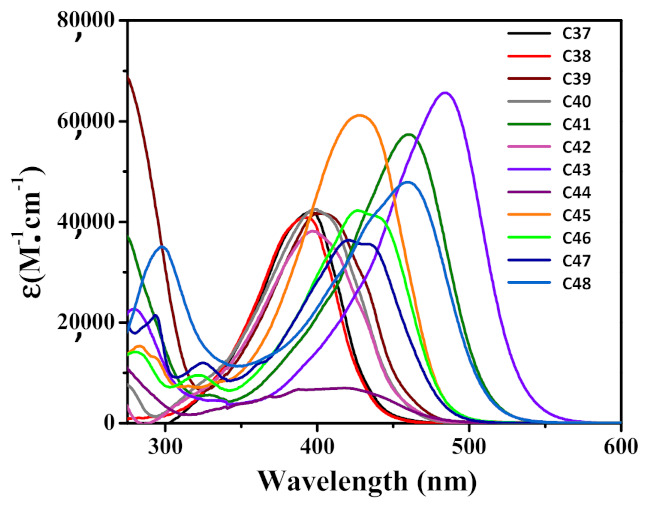
UV-visible absorption spectra of C37–C48 in acetonitrile. Reprinted with permission from John Wiley & Sons, Inc. Copyright© [207].

**Figure 19:**
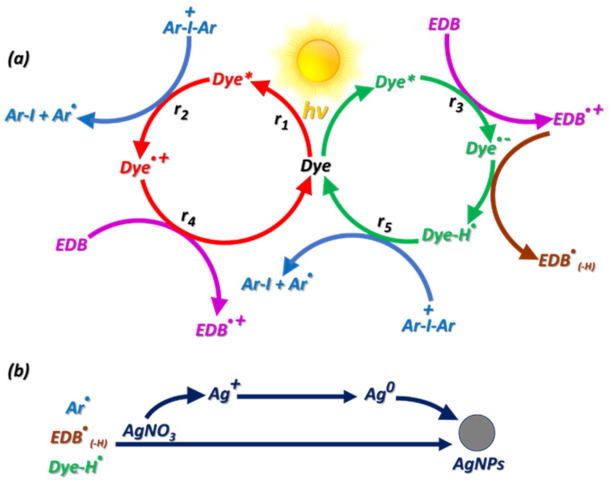
Mechanism of the photoinitiating systems enabling the formation of silver nanoparticles during the polymerization process. (**a**) catalytic cycle of polymerization (**b**) the mechanism of generation of Ag nanoparticles. Reprinted with permission from John Wiley & Sons, Inc. Copyright© [207].

Interestingly, by replacing the cyclohexanone central part with a cyclopentanone moiety in these structures, a redshift of the ICT bands could be clearly demonstrated. Thus, if an absorption maximum located at 399 nm could be found for C40, its analogue based on cyclohexanone (i.e., C12, Figure 9) showed an absorption maximum at 375 nm, blueshifted by ca. 25 nm (see Figure 18). The most redshifted absorption was determined for C43, with an absorption maximum peaking at 485 nm. To the best of our knowledge, C43 is the *bis*-chalcone exhibiting the most redshifted absorption ever used as photoinitiator of polymerization (485 nm, 65,670). The highest molar extinction coefficients could be determined for C41, C43, and C45, designed with the strongest electron-donating groups (see Table 7). Interestingly, in this work, the possibility of elaborating photocomposites via the in situ generation of silver nanoparticles was demonstrated. Indeed, by using the three-component chalcone/Iod/amine (0.1%/1.5%/1 wt%, *w*/*w*/*w*) system, phenyl radicals such as Ph•, but also Dye–H• and EDB•_(-H)_, can react with the silver salt (AgNO_3_), resulting in the reduction of the silver cation to Ag^0^ and the formation of Ag nanoparticles via the aggregation of silver atoms, in accordance with the mechanism proposed in Figure 19.

**Figure 20:**
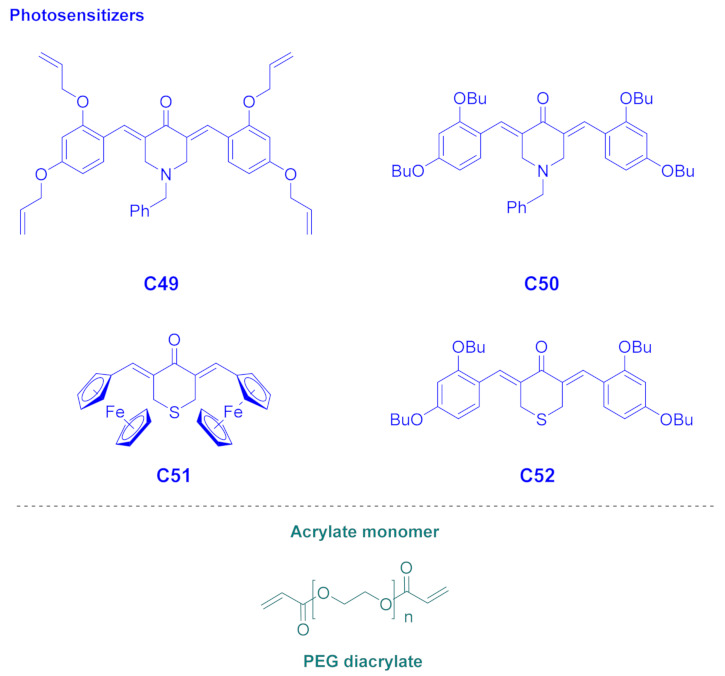
Chemical structures of C49–C52 and the acrylic monomer.

In 2021, the previous trends established concerning the influence of the substitution pattern on the photoinitiating ability of *bis*-chalcones were confirmed by a new study [112]. In this work, four *bis*-chalcones (C49–C52) containing thiopyranone or benzylpiperidinone central cores were examined as photoinitiators for the free-radical polymerization of a polyethylene glycol (600) diacrylate (PEG-diacrylate) and the FRPCP of EPOX at 375 and 405 nm, respectively (see Figure 20).

Interestingly, the presence of the ferrocene moiety in C51 enabled the drastic shift of the absorption maxima towards the visible range. Thus, absorption maxima located at 332 and 511 nm could be determined in acetonitrile for C51 (see Table 8). For the 3 other chalcones, UV-centered absorption maxima were found, peaking at 370, 380, and 369 nm for C49, C50, and C52, respectively. As anticipated, good monomer conversions could be obtained using the different three-component systems upon irradiation at 375 nm (40 mW/cm^2^). As shown in Table 7, monomer conversions ranging between 89% for C50 and 60% for C51 could be determined while using the three-component photoinitiating chalcone/Iod/amine (1.5%/1.5%/1.5%, *w*/*w*/*w*) systems, and upon irradiation of the resin for 200 s. These monomer conversions were greatly higher than that obtained with the Iod/EDB combination (49%). At 405 nm, a reduction of the monomer conversion was logically observed, consistent with a reduction of the molar extinction coefficients at this wavelength. Thus, the highest conversion was obtained with C50 (79%), whereas the lowest ones were determined with C51 and C52 (42%). In fact, a clear correlation between molar extinction coefficients and final monomer conversions could be established at 405 nm. Interestingly, at the two irradiation wavelengths, ferrocene-based photoinitiating systems proved to be the worst candidates for photopolymerization, indicating that the introduction of ferrocene to chalcone was not convenient in developing highly efficient photoinitiators. These results were confirmed by other studies devoted to ferrocene-based chalcones used as photoinitiators of polymerization [208]. Conversely, ferrocene has historically been used to initiate the cationic polymerization of epoxides [120,209]. Furthermore, photopolymerization experiments performed at 375 nm revealed the C51-based three-component system C51/Iod/amine (1.5%/1.5%/1.5%, *w*/*w*/*w*) to give the worst EPOX conversion (66%). For the three other *bis*-chalcones, EPOX conversions higher than 70% could be obtained. Here, again, the best EPOX conversion was obtained with C52 (74%), whereas this dye exhibited the lowest molar extinction coefficient at 375 nm (4800 M^−1^·cm^−1^). Therefore, the photoinitiating ability of C52 is related to its photochemical reactivity and the ease of forming *bis*-chalcone ^+•^).

**Figure 21:**
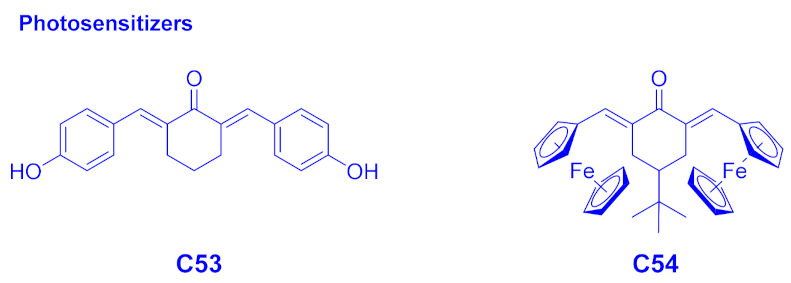
Chemical structures of C53 and C54.

It should be noted that the low photoinitiating ability of ferrocene-based dyes was confirmed with C54, which also provided lower final monomer conversions than C53 (86% vs. 95% during the FRP of PEG-diacrylate in thin films, 60% vs. 91% during the FRPCP of EPOX) (see Figure 21) [208].

**Figure 22:**
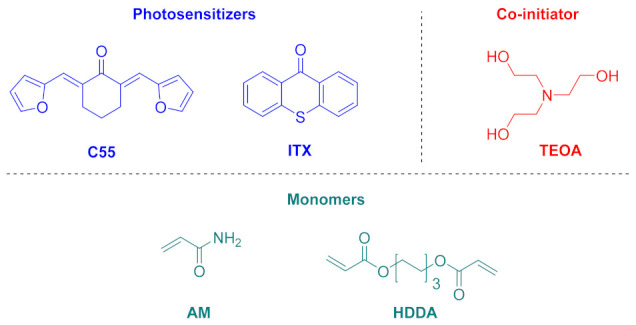
Chemical structures of C55, TEOA, and the monomer (AM).

In 2020, an elegant strategy was developed to create visible light and water-soluble photoinitiating systems from dyes absorbing in the UV range [210]. This result could be obtained by forming charge-transfer complexes between a *bis*-chalcone and a co-initiator—namely, triethanolamine (TEOA). Considering that TEOA is a water-soluble amine, a water-soluble charge-transfer complex could thus be obtained. Indeed, only few water-soluble photoinitiators are commercially available, as exemplified by 2-Hydroxy-4’-(2-hydroxyethoxy)-2-methylpropiophenone (Irgacure 2959). However, its water solubility is limited, only reaching 0.5 wt%. Many approaches have been developed over the years to convert efficient photoinitiators into water-soluble structures. Moreover, such results can only be obtained after tedious and complex syntheses [211,212,213,214,215]. Conversely, the formation of charge-transfer complexes (CTCs) is an efficient strategy to easily produce numerous CTCs without requiring extensive synthetic works [216]. Over the years, numerous CTCs have been proposed as photoinitiating systems, such as 2-isopropylthioxanthonium phenacyl hexafluoroantimonate, which could form a CTC with *N,N*-dimethylaniline (DMA) [213]. Several CTCs have also been proposed by Lalevée et al., in order to develop dual photoassisted/thermal initiating systems [206,214,217,218,219,220]. In the present work, (2*E*,6*E*)-2,6-*bis*(furan-2-ylmethylidene)cyclohexan-1-one (C55) was used as an electron acceptor for TEOA (see Figure 22).

**Figure 23:**
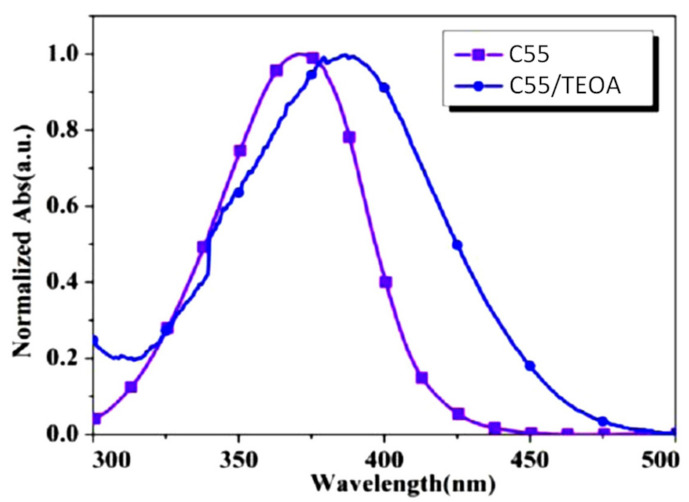
UV-visible absorption spectra of C55 and [C55-TEOA] CTC in acetonitrile. Reprinted from [210]; Copyright (2020), with permission from Elsevier.

**Figure 24:**
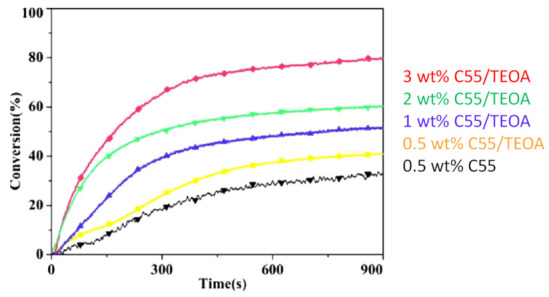
Polymerization profiles obtained upon irradiation at 405 nm (70 mW/cm^2^) of an AM resin using the [C55-TEOA] CTC as the photoinitiating system. Reprinted from [210]; Copyright (2020), with permission from Elsevier.

By mixing the two compounds in acetonitrile, a redshift of the absorption could be clearly observed, from 373 nm for C55 to 400 nm for the [C55-TEOA] CTC (see Figure 23). A saturation concentration of 5 wt% in water was determined for the [C55-TEOA] CTC. While attempting to dissolve thioxanthone (ITX) in water by mixing ITX with TEOA, ITX was determined to separate out from TEOA, indicating that the formation of a CTC between the photoinitiator and the amine was required in order to maintain the water solubility of the photoinitiator. In this context, the [C55-TEOA] CTC was used as a photoinitiating system for the FRP of acrylamide (AM) in water upon irradiation at 405 nm with an LED (I = 70 mW/cm^2^), and a concentration varying from 0.5 wt% to 3 wt% was examined. Interestingly, upon increase of the concentration, an improvement of the monomer conversion was observed (see Figure 24). Moreover, 3 wt% was determined as the optimal concentration, with an optical shielding effect occurring at higher concentrations. Indeed, when the concentration was too high, light penetration was adversely affected by an increase in the optical density, decreasing the monomer conversion.

**Figure 25:**
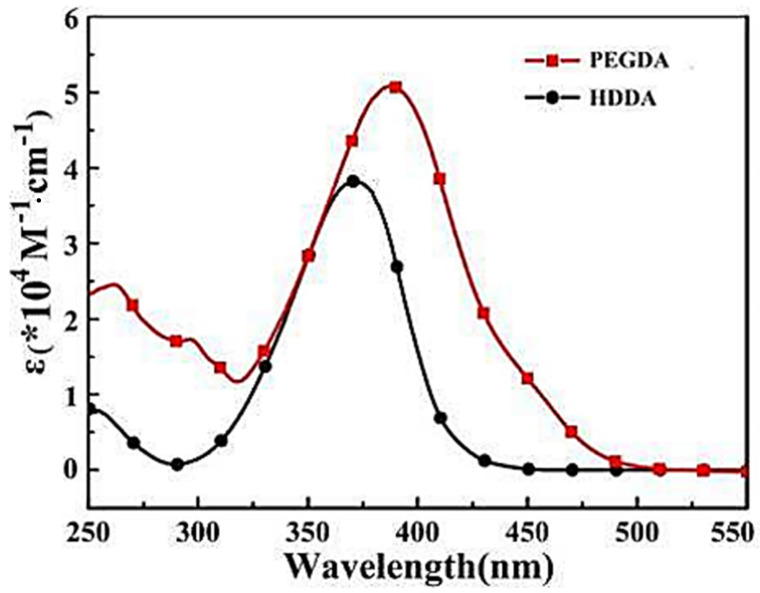
UV-visible absorption spectra of C55 in PEG-diacrylate and HDDA. Reprinted from [158]; Copyright (2019), with permission from Elsevier.

C55 is an interesting photosensitizer, since this dye was also determined to interact strongly with certain monomers; a relevant example of this was demonstrated with PEG-diacrylate [156,158]. Comparison of the UV-visible absorption spectra of C55 in PEG diacrylate and hexamethylene diacrylate (HDDA) revealed the absorption spectrum in PEG-diacrylate to be drastically redshifted compared to that observed in HDDA (see Figure 25). Thus, at 405 nm, a molar extinction coefficient of 42,800 M^−1^·cm^−1^ could be found for C55 in PEG-diacrylate, whereas a molar extinction coefficient of 35,900 M^−1^·cm^−1^ was determined in PEGDA at 365 nm. Similarly, a redshift of the fluorescence was determined for C55 in PEG-diacrylate compared to that determined in HDDA, consistent with the trend observed for the absorption.

**Figure 26:**
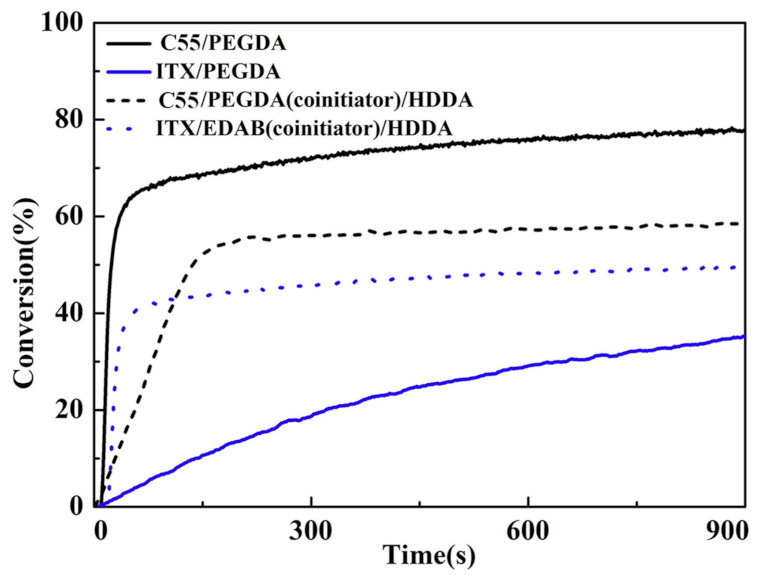
Comparisons of the polymerization profiles established for C55 and ITX used as a reference photoinitiator: C55 (0.0625% wt)/PEG-diacrylate, ITX (0.0625% wt)/PEG-diacrylate, C55 (0.0625% wt)/PEG-diacrylate (5% wt)/HDDA, ITX (0.0625% wt)/EDB (5% wt)/HDDA, LED at 405 nm, 70 mW/cm^2^. Reprinted from [158]; Copyright (2019), with permission from Elsevier.

In fact, the formation of exciplexes with protonic monomers such as PEG-diacrylate was suggested to support the modification of the absorption spectra. Interestingly, the authors demonstrated PEG-diacrylate to act as an efficient co-initiator for C55. Thus, upon irradiation at 405 nm, a monomer conversion of 80% could be obtained within a few seconds with the two-component C55/EDB (0.0625%/5% *w*/*w*) system in PEG-diacrylate, as opposed to 10% in HDDA. Replacement of EDB with PEG-diacrylate in the photoinitiating system enabled an increase in the HDDA conversion to 60% within a few seconds, upon irradiation at 405 nm of the two-component C55/PEG-diacrylate (0.0625%/5% *w*/*w*) system. The ability of PEG-diacrylate to act as a better co-initiator than the standard EDB was thus clearly demonstrated. Finally, comparison with a benchmark photoinitiator was established in order to demonstrate the potential of C55 as a photoinitiator (see Figure 26). Irrespective of the co-initiator (EDB or PEG-diacrylate), the two-component system based on C55 could clearly outperform that based on thioxanthone (ITX). Notably, in PEG-diacrylate, a 2.5-fold enhancement of the monomer conversion was observed with C55 compared to that obtained with ITX. The possibility of monitoring the polymerization process via photoluminescence measurements was also demonstrated by the same authors [157].

**Scheme 2:**
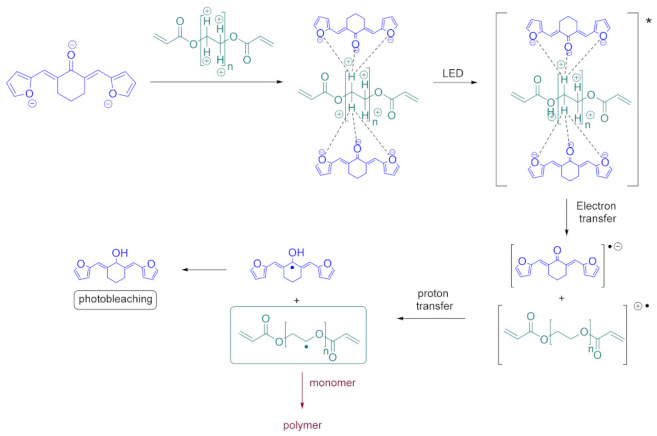
Mechanism proposed to support the high photoinitiating ability of the C55/PEG-diacrylate combination. Reprinted from [158]; Copyright (2019), with permission from Elsevier.

Finally, the mechanism depicted in Scheme 2 was proposed by the authors to support the formation of exciplexes and the ability of PEG-diacrylate to act as a co-initiator. Thus, by mixing C55 and PEG-diacrylate, hydrogen bonds can form between the oxygen atom of furan and the hydrogen atoms of the monomer. Upon photoexcitation, an electron transfer can occur between the electron-accepting C55 and the electron-rich monomer. Subsequently, by proton transfer between the radical cation of the monomer and the radical anion of C55, a radical can form on the monomer, initiating the polymerization process.

**Figure 27:**
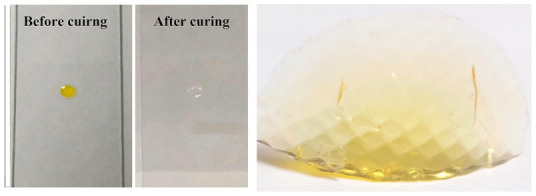
**Left**: photobleaching evidenced during the photopolymerization process; the resin before and after curing. **Right**: tridimensional structure obtained by 3D printing, exhibiting efficient photobleaching compared to the initial resin. Reprinted from [158]; Copyright (2019), with permission from Elsevier.

Interestingly, efficient photobleaching of the resin was also demonstrated with C55, and this property is actively sought for visible light photoinitiating systems, these initiating systems being highly colored. Indeed, one major drawback of visible light photoinitiating systems is the color imposed by the photoinitiator, and an extensive body of work is notably devoted to developing photoinitiators capable of bleaching upon irradiation [221,222,223]. This ability was nevertheless demonstrated during the mechanistic investigations, as well as during the different 3D printing experiments, as shown in Figure 27. Indeed, after polymerization, a complete photobleaching could be obtained, and a colorless 3D structure could be prepared.

### 3.2. Bis-Chalcones Based on Two Connected Chalcones

**Figure 28:**
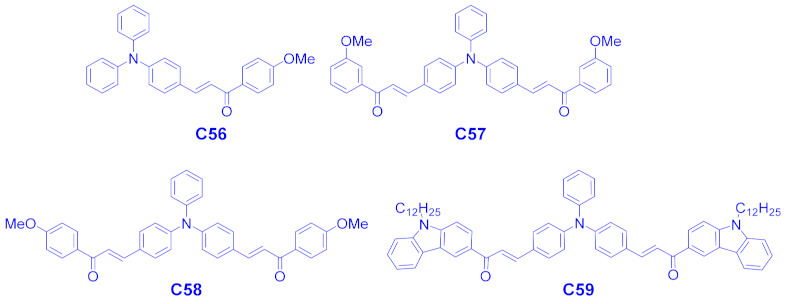
Chemical structures of *bis*-chalcones connected by mean of *bis*-aldehyde.

*Bis*-chalcones based on cyclic aliphatic ketones have recently demonstrated their promising photoinitiating abilities, from the photopolymerization kinetic viewpoint, for final monomer conversions, but also in various practical applications. Furthermore, *mono*-chalcones are also efficient photoinitiators, and numerous such structures have been examined by several research groups. In an ever-ongoing effort to further improve monomer conversion, the covalent linkage of two chalcones has been examined as a potential strategy through which to optimize polymerization efficiency. Notably, structures resulting from this covalent linkage can exhibit a redshifted absorption compared to the chalcones considered separately, provided a π-conjugation exists between the two chalcones. Jointly, by increasing the molecular weight, extractability and migratability of the structures within the polymers can be efficiently avoided. In 2020, three structures (C57–C59) based on triphenylamine, connected by means of central *bis*-aldehydes and varying in their peripheral groups, were reported by Lalevée et al. (see Figure 28) [224]. For comparison, a *mono*-chalcone C56 was also prepared.

As shown in Table 9, use of 4,4’-(phenylazanediyl)dibenzaldehyde to form chalcones C57–C59 enabled the redshifting of their absorption maxima by ca. 30 nm compared to that of chalcone C56. Indeed, if an absorption maximum at 405 nm was found for C56, the absorption maxima shifted to 430 nm for chalcones C57–C59. Interestingly, no significant influence of the peripheral groups was determined, since the replacement of a methoxyphenyl-based acetophenone by a carbazole-based acetophenone did not modify the position of the absorption maximum. Considering their absorption, the 4 chalcones were appropriate for photopolymerization experiments carried out at 405 nm, since molar extinction coefficients higher than 6000 M^−1^·cm^−1^ were determined at 405 nm. Conversely, the connection of the two chalcones by means of a π-conjugated system resulted in a significant decrease of the molar extinction coefficient. If a molar extinction coefficient of 18,740 M^−1^·cm^−1^ was measured in acetonitrile for C56, a twofold reduction of the molar extinction coefficient was determined for its analogue C58 (8540 M^−1^·cm^−1^).

Four different photoinitiating systems were used to initiate the FRP of PEG diacrylate in both thick and thin films—namely, the three-component chalcone/Iod/EDB (1.5%/1.5%/1.5%, *w*/*w*/*w*) photoinitiating system, the two-component chalcone/Iod (1.5%/1.5%, *w*/*w*) and chalcone/EDB (1.5%/1.5%, *w*/*w*) photoinitiating systems, and the chalcones alone (1.5% *w*). While no polymerization could be initiated with the chalcones alone, only thin films could be polymerized with the two-component chalcone/EDB (1.5%/1.5%, *w*/*w*) photoinitiating systems. Conversely, for the two-component chalcone/Iod (1.5%/1.5%, *w*/*w*) and the three-component chalcone/Iod/EDB (1.5%/1.5%/1.5%, *w*/*w*/*w*) photoinitiating systems, photopolymerization could be efficiently initiated both in thin and thick films (see Table 10), providing higher final monomer conversions than the two-component chalcone/EDB (1.5%/1.5%, *w*/*w*) photoinitiating systems. Interestingly, higher final monomer conversions were obtained with the two-component chalcone/Iod (1.5%/1.5%, *w*/*w*) than with the three-component chalcone/Iod/EDB (1.5%/1.5%/1.5%, *w*/*w*/*w*) photoinitiating systems, assigned to a competition with the electron-donating groups in chalcones and EDB. As a result of this, all three-component photoinitiating systems based on chalcones C57–C59 furnished lower final monomer conversions than the control experiment (blank) composed only of Iod/EDB (1.5%/1.5%, *w*/*w*). Conversely, the three-component PI systems based on chalcone C56 furnished a final monomer conversion on par with that of the blank control. Therefore, it could be concluded that EDB was inhibiting the generation of free radicals—the opposite situation to that which is commonly found for the three-component PIs. Interestingly, the three-component chalcone C56/Iod/EDB (1.5%/1.5%/1.5%, *w*/*w*/*w*) photoinitiating system and the two-component C56/Iod (1.5%/1.5%, *w*/*w*) photoinitiating system could initiate polymerization in thick films under daylight, evidencing the high reactivity of these two PIs. A polymerization ending within a few minutes could be demonstrated.

**Figure 29:**
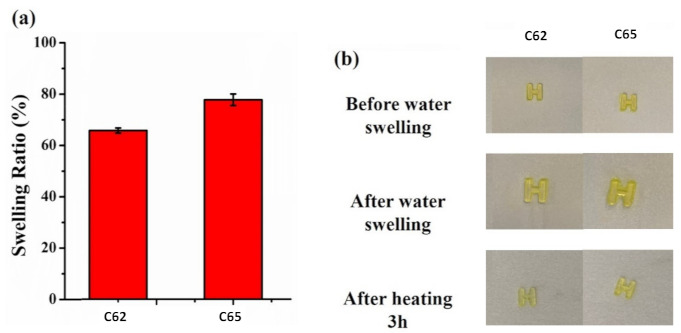
(**a**) Swelling ratios determined for PEG-based polymers prepared with C62 and C65; (**b**) pictures of the polymer 24 h after swelling and after 3 h of heating for dehydration. Reprinted from [224]; Copyright (2020), with permission from Elsevier.

Considering the high reactivity of PIs based on chalcones C56 and C59, PEG-based polymers were prepared, and due to the good affinity of PEG polymers for water, swelling experiments were carried out with polymers prepared with C56 and C59. After printing an “H” pattern in laser writing experiments using the two-component chalcone/Iod (1.5%/1.5%, *w*/*w*) system, the swelling of the PEG-based polymers was examined by immersing the polymers in water. Interestingly, a good correlation between polymerization rates and swelling ratios was determined. Thus, the highest swelling ratio was obtained for the polymers prepared with chalcone C59 (80%), due to a lower polymerization rate obtained with this chalcone compared to that obtained with chalcone C56 (92% vs. 94% monomer conversions in thick film). After swelling, a 2–3-fold enhancement of the volumes of the different 3D patterns could be determined (see Figure 29).

**Figure 30:**
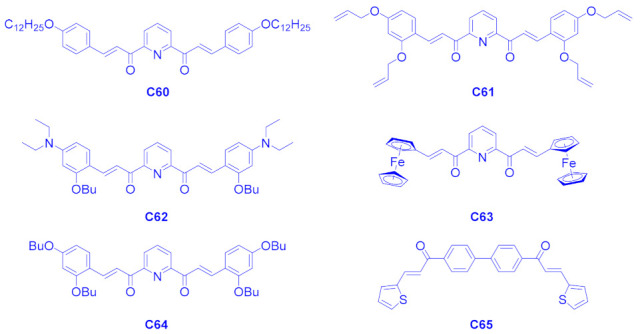
Chemical structures of *bis*-chalcones connected by mean of *bis*-acetyl spacers.

In 2021, a series of *bis*-chalcones (C60–C65) connected by mean of *bis*-acetyl spacers was examined as photoinitiators of polymerization (See Figure 30) [112]. However, compared to the previous strategy—which enabled the generation of photoinitiators absorbing until 430 nm—in the present case, the absorption of pyridine-based chalcones and biphenyl-based chalcones was UV-centered, the main absorption band being located at 350–370 nm (see Table 11). As a result of this, low molar extinction coefficients were determined at 405 nm, i.e., the emission wavelength of the light source commonly used in 3D printers. As anticipated, due to better adequation of their absorption maxima with the emission spectrum of the LED at 375 nm, higher final monomer conversions were obtained at 375 nm than at 405 nm, consistent with their molar extinction coefficients at the two wavelengths (see Table 11).

As shown in Table 12, higher monomer conversions than that obtained with the blank control could only be obtained based on Iod/EDB (1.5%/1.5%, *w*/*w*), demonstrating the crucial role of *bis*-chalcones in photoinitiation. At 375 nm, the highest monomer conversion was obtained for *bis*-chalcone C61 (95%) during the FRP of PEG diacrylate. Conversely, at 405 nm, the best conversion was determined for *bis*-chalcone C62 (95%). No direct relation between molar extinction coefficients and final monomer conversions at the two wavelengths could be established. Similarly, significant differences between *bis*-chalcones C61 and C64 could be determined in terms of final monomer conversions, even though the two chalcones only differ by alkyl chains. Here, again, the lowest monomer conversions were obtained at 375 and 405 nm for the ferrocene-based *bis*-chalcone, once again evidencing the detrimental effect of this substituent.

**Figure 31:**
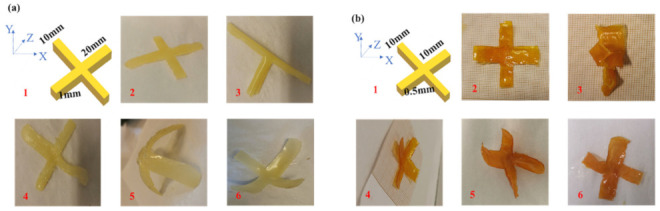
Swelling and dehydration cycles realized on 3D-printed crosses obtained using the three-component C60/Iod/EDB (1.5%/1.5%/1.5%, *w*/*w*/*w*) photoinitiating system (**a**) and using the three-component C64/Iod/EDB (1.5%/1.5%/1.5%, *w*/*w*/*w*) photoinitiating system (**b**). 1: structure of the cross; 2: cross after photopolymerization; 3: cross after one minute in water; 4: cross after 100 s of dehydration at 100 °C; 5: cross after 10 min of dehydration at 100 °C; 6: cross after 10 min at room temperature. Reproduced from [112] with permission from The Royal Society of Chemistry.

Finally, swelling experiments were carried out with *bis*-chalcones C60 and C64. Indeed, swelling experiments carried out with PEG polymers prepared with *bis*-chalcones C60 and C64 showed good swelling ratios, ranging from 60% for C60 to 70% for C64. Increases in volume as high as 160% and 143% could be determined for polymers prepared with *bis*-chalcones C60 and C65, respectively. Upon heating at 50 °C for 1 h, hydrated polymers could return to their initial appearances and volumes, demonstrating the reversibility of this hydration process. Consequently, the two photoinitiating systems were determined to be ideal candidates for 4D printing experiments. Indeed, hydration and dehydration of the hydrophilic PEG polymer can be advantageously used for shape modification. More precisely, 4D printing consists of elaborating an object of precise thickness and shape that can be modified subsequent to the polymerization process by means of an external stimulus such as heat [225,226,227,228], light [229], water [230,231,232], or other stimuli. Thus, after printing a cross with a high spatial resolution via 3D printing using the three-component chalcone/Iod/EDB (1.5%/1.5%/1.5%, *w*/*w*/*w*) photoinitiating systems based on chalcones C60 and C64, swelling and thermally induced dehydration resulted in significant modification of the shapes of the crosses. Thus, upon hydration, a complete deformation of the cross could be demonstrated. Upon heating at 50 °C, dehydration of the hydrophilic polymer could be obtained, enabling the cross to return to its initial shape (see Figure 31).

Here, again, the polymerization rate could govern the level of deformation. Indeed, due to a higher polymerization rate with *bis*-chalcone C60 compared to that obtained with *bis*-chalcone C64, less important deformations could be obtained for the PEG polymers. Conversely, for PEG polymers printed with *bis*-chalcone C64, the cross could not even be recognized after 1 min of swelling in water (see Figure 31b3). After 100 s of dehydration at 100 °C, recovery of its initial shape could be obtained.

## 4. Conclusions

**Figure 32:**
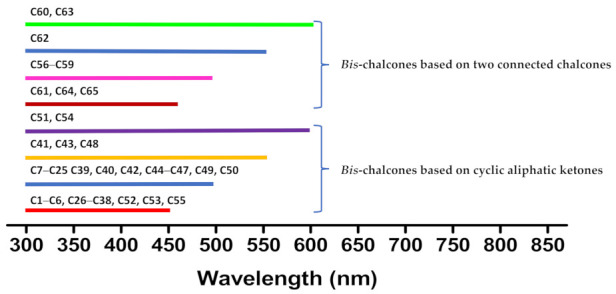
Absorption range of the *bis*-chalcones depicted in this review.

In this review, an overview of the different *bis*-chalcones reported to date as photoinitiators of polymerization has been reported. Interest in *bis*-chalcones is a recent development, following the first report mentioning the use of *bis*-chalcones as photoinitiators by Wang et al., in 2019. Since then, numerous achievements have been attained. Notably, a water-soluble photoinitiator could be obtained with C55. Photobleaching properties could also be demonstrated with this dye. Moreover, Lalevée et al. examined various *bis*-chalcones varying by their central cores and the substitution patterns of their peripheral groups. Several trends could be established. Thus, the presence of ferrocene or thiophene as peripheral groups in *bis*-chalcones was determined to drastically decrease monomer conversion. A similar effect could be evidenced with thiopyranone as the central core. Interestingly, the influence of the substitution pattern of the central cyclic aliphatic ketone was determined to only marginally impact the absorption spectra (see Figure 32), and only chalcones absorbing between 350 and 550 nm could be obtained. Among the most interesting findings, the high reactivity of chalcones enabled their access to photocomposites, and the polymerization of resins containing 20% glass fillers could be successfully achieved. Considering that the absorption of chalcones remains limited in the 350–550 nm range, future works will consist of developing *bis*-chalcones capable of absorption at longer wavelengths, enabling a higher light penetration within the photocurable resins, and easier access to photocomposites.

## Figures and Tables

**Table 1:** Light absorption properties of C1–C6 in acetonitrile.

Compounds	λ_max_ (nm)	ε_max_ (M^−1^·cm^−1^)	ε_@405 nm_ (M^−1^·cm^−1^)
C1	368	29,230	9740
C2	365	25,020	7980
C3	370	34,920	11,690
C4	368	34,200	10,130
C5	372	31,470	11,950
C6	370	29,750	10,280

**Table 2:** Final monomer conversions (FCs) for Ebecryl 40 in thick and thin films using chalcone/amine/Iod (0.1%/2%/2%, *w*/*w*/*w*), 405 nm LED, 400 s.

Compounds	C1	C2	C3	C4	C5	C6
FCs (thick films)	~30%	~24%	~94%	~24%	~90%	~25%
FCs (thin films)	~55%	~67%	~55%	~81%	~59%	~71%

**Table 3:** UV-visible absorption characteristics of C7–C19 in acetonitrile.

Compounds	λ_max_ (nm)	ε_max_ (M^−1^·cm^−1^)	ε_405 nm_ (M^−1^·cm^−1^)
C7	416	18,800	17,900
C8	405	36,200	36,200
C9	405	37,700	37,700
C10	434	30,800	23,000
C11	366	21,700	11,100
C12	375	25,200	15,500
C13	375	22,000	14,500
C14	375	23,400	15,800
C15	350	16,500	3100
C16	350	2800	900
C17	355	23,600	5300
C18	355	12,900	2700
C19	360	20,200	5700

**Table 4:** Monomer conversions obtained with the three-component chalcone/amine/Iod (0.1%/2%/2%, *w*/*w*/*w*) system, LED at 405 nm (110 mW/cm^2^), 400 s.

Photoinitiating Systems	FCs
Amine/Iod	
without chalcone	39%
C7	85%
C8	64%
C9	68%
C10	56%
C11	73%
C12	64%
C13	63%
C14	56%
C15	69%
C16	60%
C17	69%
C18	72%
C19	66%

**Table 5:** Light absorption properties of the 12 different chalcones: absorption maxima wavelength (λ_max_) as well as the molar extinction coefficients at λ_max_ (ε_max_) and at 405 nm (ε_405 nm_), respectively.

Compounds	λ_max_ (nm)	ε_max_ (M^−1^ cm^−1^)	ε_405 nm_ (M^−1^ cm^−1^)
C20	430	41,600	31,900
C21	430	45,300	35,000
C22	431	49,900	38,400
C23	430	40,000	31,900
C24	436	49,200	34,200
C25	440	43,300	30,000
C26	250	81,800	5400
C27	250	113,300	7400
C28	250	149,600	9900
C29	250	178,400	11,500
C30	250	175,900	12,000
C31	250	129,500	7800

**Table 6:** Final monomer conversions obtained during the FRP of TA upon irradiation at 405 nm with an LED for 400 s using the three-component chalcone/amine/Iod (0.1%/2%/2%, *w*/*w*/*w*) systems.

**FCs** **(in thick)**	**C20**	**C21**	**C22**	**C23**	**C24**	**C25**
	~84%	~90%	~84%	~88%	~86%	~83%
	**C26**	**C27**	**C28**	**C29**	**C30**	**C31**
	~90%	~41%	~68%	~81%	~41%	~53%
**FCs** **(in thin)**	**C20**	**C21**	**C22**	**C23**	**C24**	**C25**
	~43%	~67%	~56%	~58%	~63%	~76%
	**C26**	**C27**	**C28**	**C29**	**C30**	**C31**
	~66%	~68%	~60%	~74%	~73%	~76%

**Table 7:** Absorption characteristics of C37–C48 in acetonitrile.

Chalcone-Based Dyes	λ_max_ (nm)	*ε*_max_ (M^−1^·cm^−1^)	*ε*_@405nm_ (M^−1^·cm^−1^)	*ε*_@470nm_ (M^−1^·cm^−1^)
C37	396	41,980	37,050	270
C38	392	40,840	33,590	110
C39	400 274	41,770 69,250	41,610	2,720
C40	399	42,500	41,580	1,280
C41	460 274	57,400 37,300	23,700	53,446
C42	397	38,150	36,700	1,710
C43	485 278	65,670 22,680	16,200	59,370
C44	418 268	6,970 12,890	6800	1,600
C45	428 283	61,130 15,340	50,310	16,540
C46	427 280	42,062 14,160	32,270	14,380
C47	421 236	36,350 46,810	30,560	10,160
C48	460 298	47,620 35,060	25,300	44,400

**Table 8:** UV-visible absorption characteristics of chalcones C49–C52 in acetonitrile. Acrylate and epoxide monomer conversions determined at 375 and 405 nm.

Chalcones	λ_max_ (nm)	ε_max_ (M^−1^·cm^−1^)	ε_@375nm_ (M^−1^·cm^−1^)	ε_@405nm_ (M^−1^·cm^−1^)	PEG-Diacrylate Conversion		EPOX Conversion
					375 nm	405 nm	375 nm
C49	370	21,900	21,800	12,740	77	60	70
C50	380	28,200	28,000	19,730	89	79	72
C51	332 511	18,200 4940	5800	4420	60	42	66
C52	369	4980	4800	2550	82	42	74
blank 1	-	-	-	-	49	49	-
blank 2	-	-	-	-	-	-	45

Blank 1: Iod/amine (1.5%/1.5% *w*/*w*); blank 2: *bis*-chalcone/Iod/amine (1.5%/1.5%/1.5%, *w*/*w*/*w*).

**Table 9:** Light absorption properties of chalcones C56–C59 in acetonitrile.

Compounds	λ_max_ (nm)	ε_max_(M^−1^·cm^−1^)	ε_@405nm_ (M^−1^·cm^−1^)
C56	405	18,740	18,740
C57	430	7990	6760
C58	428	8540	7200
C59	430	10,500	9020

**Table 10:** Final monomer conversions obtained during the FRP of PEG diacrylate at 405 nm after 200 s of irradiation for thin films, and 600 s of irradiation for thick films, using the two-component chalcone/Iod (1.5%/1.5%, *w*/*w*) system and the three-component chalcone/Iod/EDB (1.5%/1.5%/1.5%, *w*/*w*/*w*) photoinitiating system.

Chalcones	Chalcone/Iod/EDB		Chalcone/Iod	
	Thin Films	Thick Molds	Thin Films	Thick Molds
C56	71%	92%	82%	94%
C57	58%	53%	77%	52%
C58	85%	17%	93%	21%
C59	85%	77%	93%	92%
blank	50%	93%		

**Table 11:** UV-visible absorption properties of *bis*-chalcones C60–C65 in acetonitrile.

Chalcones	λ_max_ (nm)	ε_max_(M^−1^·cm^−1^)	ε_@405nm_ (M^−1^·cm^−1^)	ε_@375nm_ (M^−1^·cm^−1^)
C60	347	23,100	6800	16,500
C61	364	22,700	10,070	21,500
C62	430	38,900	26,420	8500
C63	330	19,800	2960	4000
C64	370	24,600	13,670	24,000
C65	350	49,300	2220	24,500

**Table 12:** Final monomer conversions determined during the FRP of PEG diacrylate using the 5%/1.5%/1.5%, *w*/*w*/*w*) photoinitiating systems shown in Table 1, at 375 and 405 nm.

**LED@375 nm**							
Chalcones	C60	C61	C62	C63	C64	C65	Blank
FCs	86	95	80	64	89	82	49
**LED@405 nm**							
Chalcones	C60	C61	C62	C63	C64	C65	Blank
FCs	62	83	95	53	91	62	49
